# Xurography as a Rapid Fabrication Alternative for Point-of-Care Devices: Assessment of Passive Micromixers

**DOI:** 10.3390/s16050705

**Published:** 2016-05-16

**Authors:** J. Israel Martínez-López, Mauricio Mojica, Ciro A. Rodríguez, Héctor R. Siller

**Affiliations:** Tecnológico de Monterrey, Eugenio Garza Sada 2501 Sur, 64849 Monterrey, N.L., Mexico; israel.mtz@itesm.mx (J.I.M.L.); mojica@itesm.mx (M.M.); ciro.rodriguez@itesm.mx (C.A.R.)

**Keywords:** xurography, lamination, rapid fabrication, micromixer, splitting and recombination, SAR, ASAR, in-plane, low-cost, microfluidics, Point-of-Care

## Abstract

Despite the copious amount of research on the design and operation of micromixers, there are few works regarding manufacture technology aimed at implementation beyond academic environments. This work evaluates the viability of xurography as a rapid fabrication tool for the development of ultra-low cost microfluidic technology for extreme Point-of-Care (POC) micromixing devices. By eschewing photolithographic processes and the bulkiness of pumping and enclosure systems for rapid fabrication and passively driven operation, xurography is introduced as a manufacturing alternative for asymmetric split and recombine (ASAR) micromixers. A T-micromixer design was used as a reference to assess the effects of different cutting conditions and materials on the geometric features of the resulting microdevices. Inspection by stereographic and confocal microscopy showed that it is possible to manufacture devices with less than 8% absolute dimensional error. Implementation of the manufacturing methodology in modified circular shape- based SAR microdevices (balanced and unbalanced configurations) showed that, despite the precision limitations of the xurographic process, it is possible to implement this methodology to produce functional micromixing devices. Mixing efficiency was evaluated numerically and experimentally at the outlet of the microdevices with performances up to 40%. Overall, the assessment encourages further research of xurography for the development of POC micromixers.

## 1. Introduction

Development of viable and dependable Lab-On-a-Chip technology for unindustrialized countries is one of the current goals of the scientific community. The availability of Point-of-Care (POC) diagnostics is particularly relevant for places where infrastructure is limited and technological resources are scarce. The detection of illnesses in early stages can change dramatically the prognosis of individuals and POCs can be an instrument to improve the current detection processes. Furthermore, early detection can translate into significant savings by allowing treatments at earlier stages of illnesses, and prevent the spread of infectious diseases. A criterion called “ASSURED” which stands for affordable, sensitive, specific, user-friendly, rapid and robust, equipment-free or minimal equipment and deliverable to end-users has been coined from UNDP-World Bank-WHO Special Programme for Research and Training in Tropical Diseases. The development of Lab-On-a-Chip POC devices in challenging environments poses special design criteria: in developing countries, devices require to perform reliably under constraints of low cost, the absence of trained staff, lack of electricity, poorly equipped laboratories facilities and with limited access to refrigeration and storage [1,2].

Rapid fabrication is a family of manufacturing techniques in which a part or a physical component is fabricated in short periods of time comparatively to more common and known manufacturing processes. It fulfills the fabrication requirements for some applications and can even produce parts that cannot be made using conventional methods [3]. Incorporation of rapid fabrication technology for microfluidic devices is still an ongoing process to be consolidated. The shift in material preference from semiconductors and glass to polymers and paper had increased the chances of expanding the reach of microfluidic Lab-On-a-Chip devices thanks to their inherent lower material costs. Recent literature review articles discuss major advancements in the development of this technology for sensing, cell culture, and particle sorting and classification. However, many of the advances employing microfluidics are still limited to laboratory prototypes that require specialized staff and access to clean room equipment to operate. For that reason, their application is still unavailable for developing countries. The most widely approach for microfabrication so far has been silicon micromolding which relies on the fabrication of structures through photolithographic processes [4]. Conventionally this implies multiple operation procedures that require supervision by specialized personnel under laboratory facilities, with equipment as spin coaters and ovens, and with supplies of photosensitive materials for the development of thin structures with resolutions of a few micrometers. These structures can be used as a final device or to replicate it through casting, stamping or injection molding. Rapid fabrication techniques have recently approached the finesse of conventional microfabrication; for example micro-milling [5] or laser ablation [6] have been able to produce quickly devices within resolutions around 50 to 100 microns. However, these techniques still require access to relatively expensive equipment and tools.

Compared to other emerging low-cost benchtop technologies like 3D printers or laser cutters, xurography and lamination of thin films remain as an attractive alternative for POC environment considering the communication restrictions. Thin film materials in rolls or sheets can be transported more easily than liquid based reagents like polydimethylsiloxane (PDMS) or resins used for additive manufacturing process in stereolithographic processes. Moreover, typically these materials are restricted by expiry dates and the need for temperature and light protected environments during transportation and stocking.

## 2. Xurography and in-Plane Micromixers for POC Devices

Around a decade ago the works of Bartholomeusz *et al.* and Treise *et al.* lead the groundwork of xurography as an innovative microfabrication technique [7,8]. This equipment relies on removing material from thin films through a cutting blade controlled by commercially available systems that were originally intended for production of large advertisement signs. Simplicity and quickness in operation, without requiring special facilities for their operation (*i.e.*, clean rooms or supplementary specialized laboratory equipment for photolithographic processing of materials) are advantages derived from the manufacturing technology.

Ten years ago, typical professional quality xurography equipment cost around $4000 USD. Nowadays, the cost of this type of equipment has dropped by at least 50%. Furthermore, there is newer desktop-based equipment with comparable resolution precision (5 to 10 μm) and improved portability that can be found for around $150 USD. The significant cost reduction is related to the change in the scale of the cutting area as new devices are intended for cutting 9 or 12 inches wide materials rather than full sized posters for advertisements or decorations within the range. This transition has not meant a sacrifice in the resolution of the cutting system and instead has led to the appearance of more user-friendly interfaces and improved portability. Lamination or stacking several layers of thin films has been proven to be an effective way to produce more complex geometries [9,10].

Despite the fact that xurography has not enjoyed a prominent position in academic research, some notable applications are worthy of citation. A research group at the University of Utah worked on a comparison between double-sided tape devices made through xurography and glass, and devices manufactured through lithography and glass etching [11]. They concluded that both techniques performed with similar signal-to-noise ratio but with considerable time and money savings with the former. Kolekar [12], from the same institution, conducted an extensive experimental study of the fluid flow characteristics of microchannel systems fabricated by xurography from double-sided adhesive between two glass plates with inlets and outlets ports under a clamping system composed of a PDMS cushion and a polycarbonate (PC) clamp sheet. De Santana *et al.* recently evaluated the feasibility of xurography to fabricate masks in a hydrofluoric acid (HF) etching process for the development of electrophoretic devices. Glass microchannels fabricated by xurography provided resolved, well-defined, and highly repetitive electrophoretic separation platforms [13]. Sundberg *et al.* developed a spinning disk platform for microfluidic digital polymerase chain reactions by patterning spiral designs on 125 μm thick PETG sheets and bonding them thermally to create a laminated three-layer PCR thermocycler [14]. Kim *et al.* recently assembled a seven-layer microfluidic electrochemical biosensor cartridge by combining double-sided tapes, gold-coated PET films, PET films, and acrylic sheets. While in this case the microfabrication was assisted by CO_2_ acrylic laser cutting and Au sputtering, the researchers demonstrated that it was possible to build an electrochemical immunoassay device within clinical norms recurring to a rapid fabrication technique that enabled the design, fabrication and testing of a new device on a single day [15].

Table 1 shows that xurography and lamination have been employed as a complementary step to conventional manufacturing techniques (heat treatment, laser cutting, thin-film development) to develop microfluidic devices. Nevertheless, the selection of expensive materials or process hinders the impact of these designs for POC implementations.

Component-based microfluidic R & D approach has shown to be slow and expensive [16]. Regardless of the function (transport, metering, mixing, concentration or separation) Lab-On-a-Chip devices are complex since they imply particular design decision regarding manufacture material and methodology, ancillary technology for driving the reagents, transducers, and biotechnology. Mutual compatibility among these factors is a challenge to be considered in terms of fully functional devices. The gap between academic projects and viable deployable POC technology arises from neglecting the importance of integration aimed at commercialization [17]. Furthermore, successful operation of devices requires the capacity to perform all the operations together and at once, not separately, in an automated manner [1,2,17,18].

Micromixing is one of the desired functions in POC devices because it can play a significant role when the characteristic time scale of a reaction involved has the same magnitude or is smaller than the time scale of the mixing process [19]. Micromixers are microfluidic system components that can be used for the homogenization of sample reagents. Passive micromixers do not require any external energy source and mix the fluids by a special geometry that creates a flow field. By means of splitting, stretching, folding and breaking the flow this can lead to a mixing process with an exponential decay of the characteristic length over which diffusion acts [20]. There is an extensive background literature on passive micromixers that draws upon three-dimensional features along the flow chamber to create secondary flows like the slanted grooved micromixer [21,22] (SHM) or the staggered herringbone micromixer [23] (SHM). These barrier embodied microdevices have some manufacture and performance limitations; the three-dimensional features tend to require complex microfabrication methods; fluidic resistance and hence pressure requirements for pumping system tend to increase within these complex geometries.

The simplest passive micromixer is the T-mixer where two separate fluids are brought into contact from opposite directions and then leave through a channel that is perpendicular to the inlet channels [24]. However mixing occurs only proximate to the junction, the flow in a straight channel later becomes laminar. Split and recombination micromixers (SAR or ASAR for asymmetric designs) rely on accelerating the diffusive transport process by repeatedly forcing contact of multiple parallel fluid streams and increasing the interfacial area.

Splitting and recombination devices that employ in-plane micromixing emerged in a later stage of microfluidic device development (the middle of the 2000s) when PDMS micromolding was already a standard procedure in research. While some earlier works reported development with other materials such as COC [25] or SEBS [26], recent developments have focused on this elastomer. PDMS is the most popular elastomer due its ability to be cast with nanometric resolution, relative inexpensiveness and because it can be irreversibly bonded to other materials such as glass or other polymeric films [27,28]. While differences among the designs of in-plane micromixers are noticeable (ranging froms rhombic [29,30,31], modified Tesla [25,32,33], circular setups [26,34,35] or logarithmical [36] shapes), the geometrical features, with channels from 150 to 500 μm, are clearly within the manufacturing range of PDMS manufacturing via photolithography or molding. However, this selection implies a need for supplementary equipment and tools for bonding and sealing the devices and incorporating other materials (PTFE, silicon, or glass) to interface with the device.

There has been in the literature little consideration regarding commercialization or pursuit of the ASSURE criteria. The increasing gap between methods and materials used in research and commercialization [37] remains among the challenging conditions for POC manufacture.

For this work, development of a passively driven technology based on splitting and recombination was pursued for the following reasons: first, micromixing has shown potential as a low-cost sensitivity enhancer for biosensing micro-devices [38,39,40]. Second, the performance of passive micromixers depends directly on the geometrical features of the microchannels and hence represents a reasonable proof of concept for the presented methodology. Third, SAR microdevices are inherently less complex than three-dimensional flow geometries like barrier embodied setups and could be more easily integrated with other devices to perform more complex functions. Finally, by eschewing nonessential tools and equipment a step toward the development of low-cost deployable POC devices can be taken.

After examination of the literature regarding xurography and micromixers, the authors did not encounter any previously reported implementations of xurography for manufacturing either micromixing or SAR devices. Hence, this assessment aims to develop the proposed methodology by addressing the limitations in material availability and cutting precision without the support of potentially unavailable tools in the POC setting. Despite the abundance of prototypes involving xurography for varied functions (see Table 1), bonding of layers of the devices has been mostly carried out by thermal or chemical adhesion, or by the use of framing or clamping systems. For this project we have considered a simple and affordable assembly methodology based on intercalating hard and soft thin layers of materials to conform the micromixer.

Implementing the methodology above using a geometry-dependent design requires the characterization of the cutting process within the range of the micromixers width channels to evaluate and optimize the manufacturing process. To allow researchers and developers of POC devices to exploit the insights derived from our assessment, we have selected a well-known design (T-micromixer) as a reference for cutting and assembly. Then, more complex SAR designs (within the effects of changes derived from the manufacturing process) were numerically and experimentally evaluated.

## 3. Materials and Methods

### 3.1. Rapid Fabrication Process

A Graphtec CE5000-60 (Graphtec America, Irvine, CA, USA) high precision cutting plotter was used to fabricate the different layers of the microdevices. Standard commercial Arlon vinyl sheets (Placentia, CA, USA) were used for the manufacturing assessment (this provider was selected considering the availability of a worldwide distributor network). Typically, these machines are loaded with sheet rolls of different widths ranging from 38.1 to 152.4 cm. Testing were made over two different polymeric films for the gasket layer or Layer 0, a vinyl with a commercial name of 4500 CalPlus with 75 μm of thickness and 122 cm of width (blue color) and a vinyl with a commercial name of Premium cast 2100, with 50 μm of thickness and 61 cm of width (black color). Commercial acetate sheets transparency foils were used to pattern intermediate layer (Layer 2; 21.59 cm × 27.94 cm). The thickness of the acetate sheets was found to be variable among the stock and was examined using confocal microscopy prior to experimentation. Devices were sealed using a CalPlus 5000 transparent polymeric film (Layer 3). Transfer paper (American Biltrite, Wellesley Hills, MA, USA) was used to hold the patterned layer temporarily. Tweezers were used to remove the spare vinyl film. Commercial acrylic glass sheets (50 mm × 50 mm × 2.5 mm, Plaskolite, Columbus, OH, USA) were manually divided into square substrates using an acrylic cutting tool. A Computer Assisted Design (CAD) program was used to design the layers and to export their geometrical features in the form of DXF file de facto standard extension to the cutting machine software (Graphtec Design Studio) for patterning the Layers 1–3. The cutting tool used for this work was a standard carbide cutting tool model CB09U with a cutting diameter of 0.9 mm and a cutting angle of 45°. Feed rate was set to 1 cm/s and the cutting load was varied as indicated in Table 2. Distilled water and food colorants were used for visual inspection. The blue dye was composed of water, glycerin and corn syrup. The red dye was composed of monopropylene glycol and artificial colors. Microdevice characterization was carried out with a SV6 stereoscopic microscope (Carl Zeiss Microscopy, Cambridge, England) to measure the microchannel features (see Table 2). An Axio CSM 700 confocal microscope (Carl Zeiss Microscopy, Cambridge, England) was employed to determine the depth of the microdevices during the different steps of the lamination process.

### 3.2. Patterning Parameter Optimization (Layer 1)

To assess the impact of the cutting parameters on the dimensional variability of the microchannels, we considered two different cutting loads and two different cutting passes settings in an experimental array with three replicates for each setup (Table 3). These setup values were selected following previous literature and preliminary patterning and cutting experimentation.

### 3.3. Testing Reagents and Materials for Microdevices

**Figure 1:**
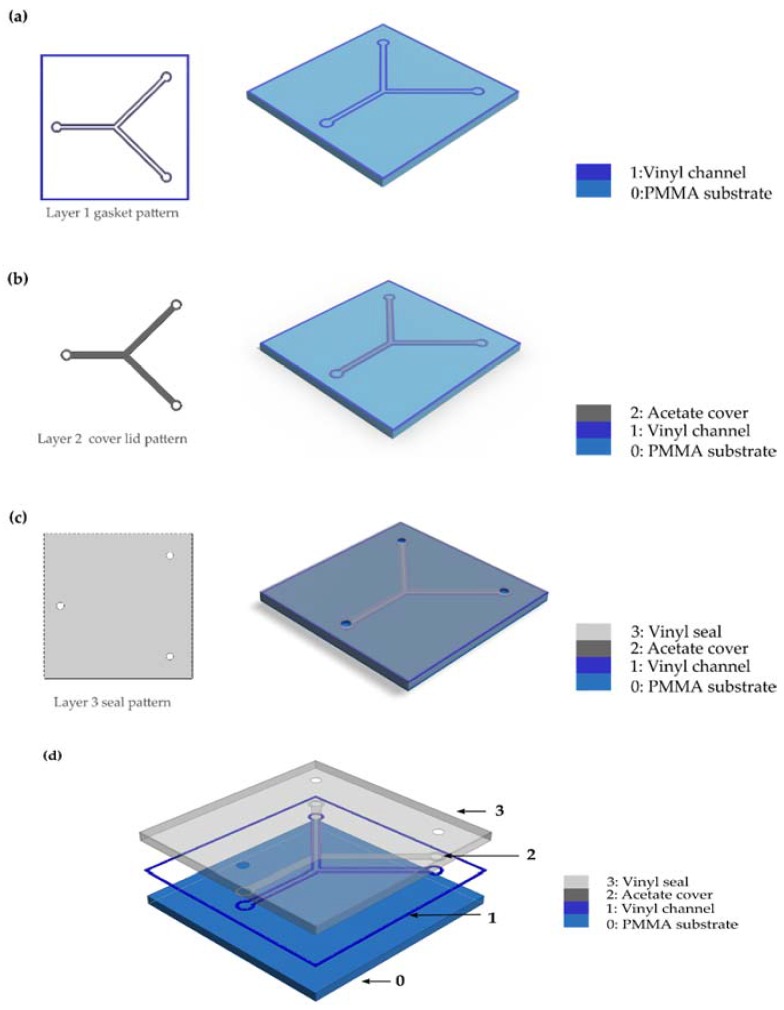
Rapid fabrication methodology based on xurography patterning and lamination; (**a**) Layer 1 patterning and adhesion to substrate; (**b**) Layer 2 patterning and alignment; (**c**) Layer 3 patterning, alignment, and final assembly; (**d**) Exploded view of a sample microdevice.

Figure 1 shows the rapid fabrication methodology assessed: a design made on CAD software is exported to a proprietary software of the cutting plotter (Graphtec Studio), a pattern of the vinyl material is made (Layer 1) following the geometric features contained in the DXF file. 

Assisted by transfer paper, the design is relocated from the original underlying paper substrate into a PMMA substrate (Layer 0), regions of the channel are removed to define the walls of the microchannel onto the first phase of the assembled device (Figure 1a). A second layer is imported in the DXF extension to the software and patterned (Layer 2) into the acetate sheet to provide enclosure to the channels defined in the previous step (Figure 1b). Then, the inlets and outlets of the microchannel are patterned in another vinyl sheet design (Layer 3) and wrapped to form an integrated device (Figure 1c). Finally, the device is comprised of four layers (see Figure 1d).

The geometry selected for the evaluation of the patterning quality of the manufacturing process was a T-micromixer (Table 3 summarizes the nominal geometric features and Figure 1a shows the shape of the device).

### 3.4. Cutting Portability Test

To assess if the current methodology is applicable with other equipment and setups (details are shown in Table 4), a T-micromixer (w = 750 μm) pattern was made employing a new (unused) blade, with an alternative cutting angle (60°) and using desktop portable plotter on 4500 CalPlus thin film material. These datasets were collected using the methodology described above (Section 3.1.).

### 3.5. Proof of Concept: SAR and ASAR Passive Micromixers

#### 3.5.1. Split and Recombine Micromixer Design and Rapid Fabrication

Implementation of the rapid fabrication methodology studied in this article was assessed in the manufacturing process of a passive micromixing device, in addition to the T-micromixer used for xurography evaluation. The implemented design is based on previous work done by Ansari *et al.* [34,35] where micromixing is achieved by splitting the main channel into sub-channels and recombining them after a certain distance.

**Figure 2:**
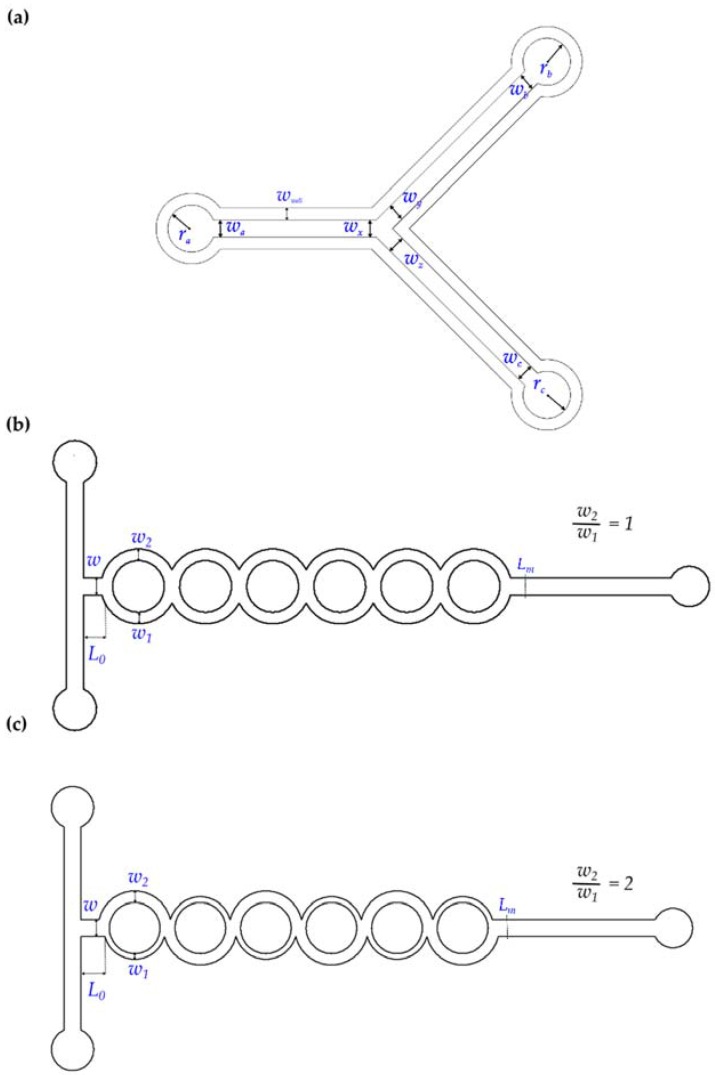
Schematic diagram; (**a**) T-micromixer (45° angle) shaped pattern cutting references (see Table 2 for details); (**b**) Balanced split and recombine (*w_2_/w_1_* = 1) micromixer (SAR); (**c**) Unbalanced (asymmetric) split and recombine micromixer (*w_2_/w_1_* = 2) (ASAR).

For this work, scaled up versions of the aforementioned designs (250% from the original dimensions) were developed from scratch following the methodology described in Figure 1. Figure 2b,c describe the design where the main channel with a fixed width (*w*) is split into two sub-channels (*w*_1_ and *w*_2_) and then recombined after a certain distance. Two types of splitting and merging micromixers were manufactured: a balanced collision case whereas the sub-channels had the same width (Figure 2b; *w*_1_ = *w*_2_ = 750 μm) and an unbalanced collision setup (Figure 2c; *w*_1_ = 250 μm, *w*_2_ = 625 μm). Axial length from the input (*L*_0_) was set to 1250 μm for a six segment splitting and recombination setup. The output of the micromixer (*L*_m_) was considered at 22,750 μm from the input. The depth of the micromixer devices (*d_m_*) was approximately 100 μm according to the measures taken with the confocal microscope.

For examination of the micromixing performance of the microdevices, red and blue food colorant dyes were diluted in distilled water and pipetted over the inputs of the devices to propitiate a passively driven flow. For both cases, red and blue solutions were introduced simultaneously into the device by pipetting 100 μL droplets over the inputs. Distilled water and a standard compressed air duster (Office Depot, Boca Raton, FL, USA) were used to clean the devices before and after using them.

#### 3.5.2. CFD Analysis

The flow and mixing in the micromixer were numerically analyzed by solving three-dimensional continuity and Navier-Stokes equations. Comsol Multiphysics 4.3b modules were employed, specifically the Laminar Flow and the Diffusivity of Target Species ones. The flow was assumed at steady state and incompressible.

Parameters were set considering water properties as follows; density (ρ) was considered in 9.998 × 10^2^ kg·m^−3^ and the dynamic viscosity (σ) in 1.01 × 10^−3^ kg·m^−1^∙s^−1^ according to temperature conditions at 20 °C [22]. Concentration was set to 1 mol/m^3^ at the upper inlet to evaluate the degree of mixing the while the other was set to zero. Concentration was assessed along the cross-section of the channel and at the output. Volumetric flow rates were equal for both inlets and varied for evaluating the performance of the micromixer under different Reynolds numbers. Since the diffusivity properties of the food dyes were not available from the device, their properties were estimated from the values reported in the literature from for their principal components (monopropylen glycol for the red dye and water for the blue dye). Hence, the diffusion coefficient of the liquid introduced at the superior inlet (*D_c_*) was considered 1.24 × 10^−9^ m^2^·s^−1^ following properties reported before in literature [41,42].

For evaluation of the mixing eficciency at the output of the micromixer, cross-sectional planes were defined and evaluated within regular point grids along the output channel starting at the *L_m_* length and subsequently every 500 μm up to 4500 μm.

#### 3.5.3. Evaluation of Experimental Micromixing Efficiency

The SAR and ASAR devices microfabricated with xurography and lamination were evaluated experimentally using images captured with a digital camera using Image Processing Lab (Aforge.NET) [43] and ImageJ [44] for image processing. Evaluation windows (47 × 215 pixels) were defined every along the output of the channels to extract the image RGB mean and variance values. The variance of one of the components (Red) was selected along with maximum variance baseline (σ_max_) established from a completely unmixed image reference.

## 4. Results and Discussion

### 4.1. Rapid Fabrication through Xurography

To recapitulate the rapid fabrication process presented here, Table 5 summarizes the composition of the layers and their specific functions used for the geometric evaluation. Assembly of each device could be done by a single person in a short period assisted by simple manual tools (scissors, tweezers and a cutting pad). For example, patterning of Layers 1 and 3 took around 4 minutes for each device. Stacking material to produce a microdevice was employed successfully to fit the requirement of the device with the proper materials: PMMA from the Layer 0 provided mechanical stiffness required for layer stacking. Vinyl from Layer 1 could be patterned easily and quickly for the development of the microfluidic channel walls, and the ceiling of the device could be made with the acetate sheet with the additional benefit of providing structural support for the covering transparent Layer 4. Alignment between Layers 2 and 3 proved to be critical in the overall function of the device; mismatches could potential produce inaccessibility to the inlet or outlet ports.

**Figure 3:**
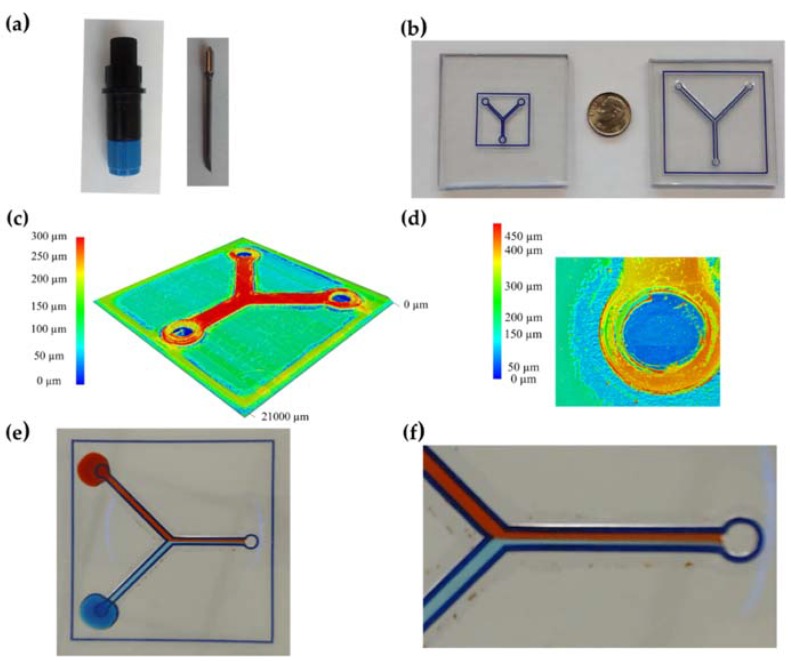
Rapid fabrication of T-micromixer (45°); (**a**) Cutting plotter holder and tool; (**b**) Photos of *w_nom_* = 200 μm device (left) and *w_nom_* = 750 μm (right); (**c**) 3D mapping of a *w_nom_* = 200 μm device (cases I to IV); (**d**) 3D mapping through confocal microscopy at an inlet of a *w_nom_* = 750 μm device (cases V to VIII); (**e**) T-micromixer sealing test; (**f**) Detail of laminar flow performance on a T-micromixer device.

Figure 3a,b show the cutting tool used and some examples of fabricated T-micromixers. Confocal microscopy measurements showed that Layer 1 was consistently deeper than nominal (100 μm), however, channel flow cell formed between Layer 0 and 1 remained equal (flattening below 5%) to the nominal depth (*d_nom_*) after lamination, hence, the depth of the vinyl Layer 1 before lamination could be approximated to the depth of the layer before cutting. Sealing layers 2 and 3 (Table 5) neither displayed significant changes due stacking. Figure 3c shows a confocal microscopy image of a *w_nom_* = 200 μm full device (cases I to IV) with an overall depth of the device (not considering the PMMA substrate) of approximately 300 μm (Layer 1 ≈ 100 μm, Layer 2 ≈ 100 μm, Layer 3 ≈ 100 μm). Figure 3d shows the three-dimensional image obtained with the confocal microscopy of a *w_nom_* ≈ 750 μm device (cases V to VIII) with an approximate the overall device depth of 380 μm (Layer 1 ≈ 100 μm, Layer 2 ≈ 100 μm, Layer 3 ≈ 180 μm). Planar uniformity along the device was found between the manufactured devices.

Cutting plotter settings had shown to be necessary for determining minimum feature size [7]. If the cutting feed rate is too fast, the features will not form well due to tearing and features might be missed where there are abrupt changes in direction. Cutting force must be set to a value where the material is cut but controlled that the backing of the material is only scored.

Patterning the designs for the Cases IV to VIII showed initially deviation errors prohibitively high for any practical application (above 30%). Seemingly, the cutting blade was not able to follow the software directions quickly enough. Considering the feed rate was already set to the minimum value, we implemented a compensation on the cutting references (see Table 6). 

These values were determined by averaging the deviation error from five devices patterned with the original nominal values. Figure 3e,f show a functional T-micromixer device (w_nom_ = 200 μm) developed through xurography and lamination with the aforementioned dimensional compensation under a sealing test using food colorants for visualization where laminar flow is presented for passively driven flow (video of a sealing test can be found on the Supplementary Materials online). For Cases I to IV devices, the methodology aforementioned was applied without any compensation.

### 4.2. Microdevice Quality Assessment

**Figure 4:**
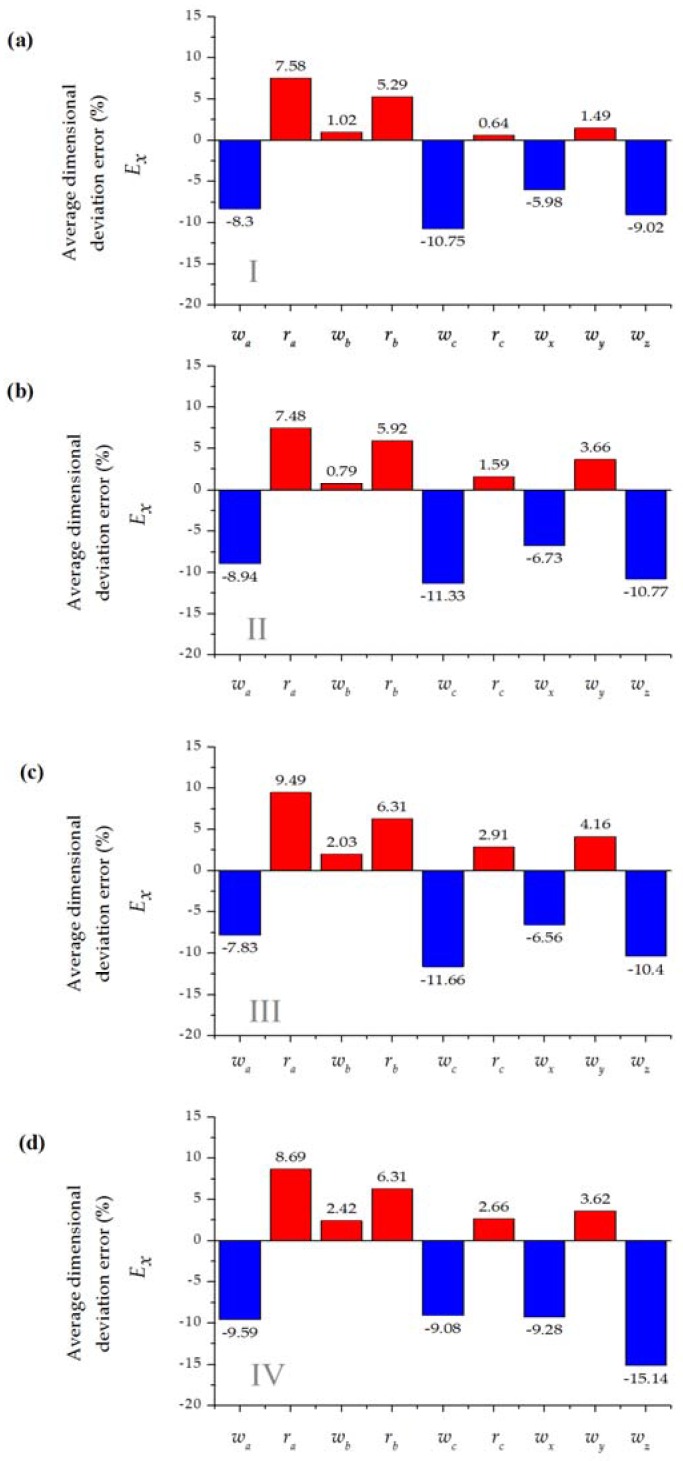
Average dimensional deviation error (%) for a *w_nom_* = 750 μm microchannel T-micromixer design with three replicates (see Table 3 for details, red represents overcutting and blue undercutting; (**a**) Setup I; (**b**) Setup II; (**c**) Setup III; (**d**) Setup IV.

**Figure 5:**
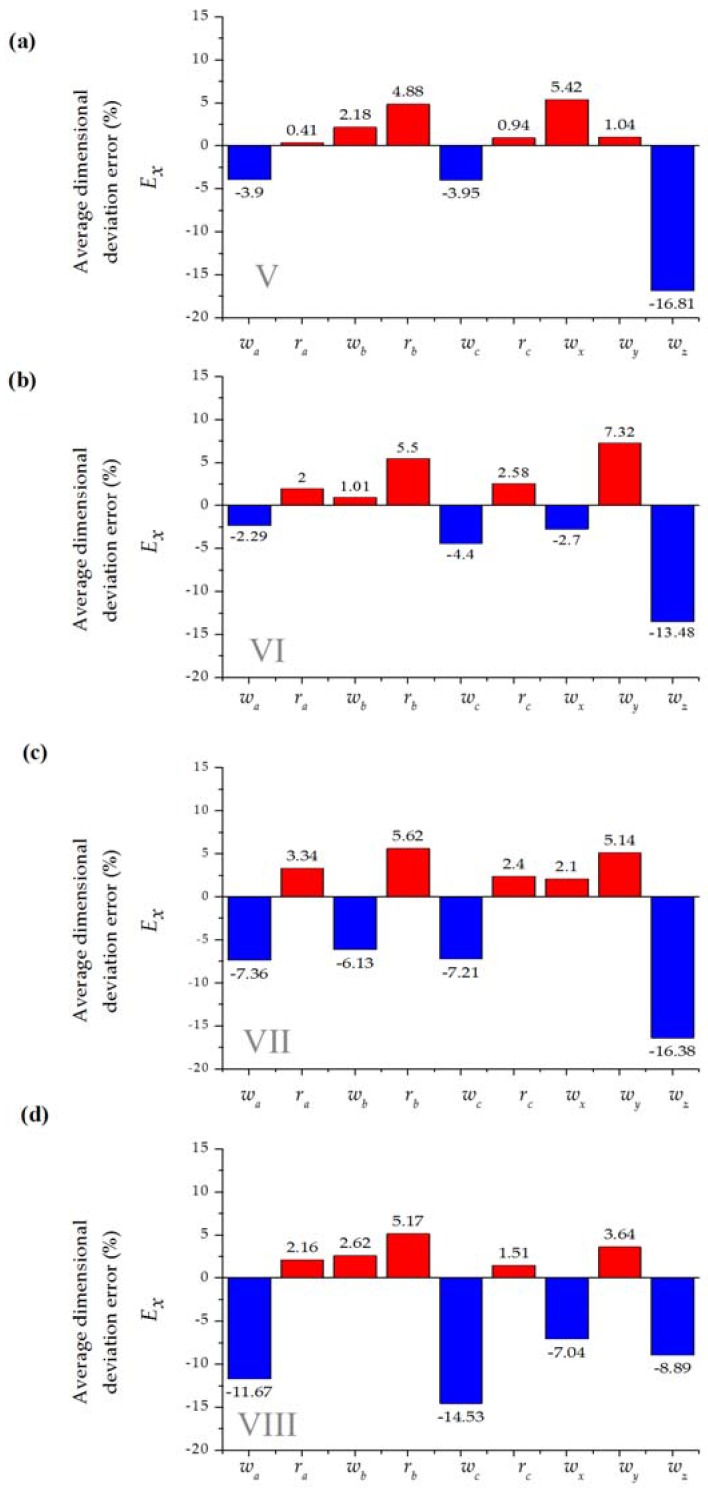
Average dimensional deviation error (%) for *w_nom_* = 200 μm microchannel T-micromixer design with three replicates (see Table 3 for details, red represents overcutting and blue undercutting); (**a**) Setup V; (**b**) Setup VI; (**c**) Setup VII; (**d**) Setup VIII.

Figure 4 and Figure 5 show the average dimension deviational error for the different fabricated geometric features. 

Measurements showed undercutting or overcutting variability regardless of the material depth, the number of cutting passes or pressure conditions. Specifically, rounded features present overcutting while linear features present undercutting in a consistent way. This process behavior may be an effect of the interpolation direction that the cutting tool performs during each cutting pass and as a result of a lack of a width-of-cut compensation algorithm in the machine controller.

**Figure 6:**
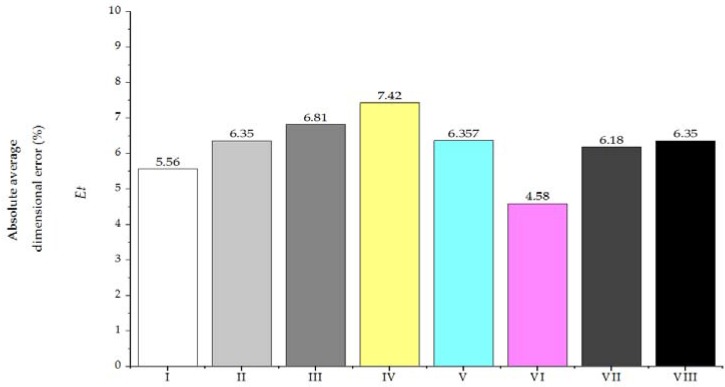
Absolute average dimensional error *E_t_* (%) for several setups (see Table 2 for details).

Figure 6 comprises the average absolute error among the experimental patterning setups. While this work is not intended as a thorough analysis of cutting parameters, we found overall better performance for the thicker blue material. For the two sizes of the microchannels (*w_nom_* = 700 μm and *w_nom_* = 250 μm), it was possible to develop devices with average deviations (*E_x_*) below 17% (Figure 5), which is considerably higher than those reported for systems based replication molding polymer (about 2%) [45]. Manufacture of integrated devices based on xurography carries errors derived by manually driven operations during the lamination process.

Figure 6 describes a general evaluation of the assessed parameters considering the absolute average dimensional error (*Et*) which comprises the under and overcutting errors presented in Figure 4 and Figure 5 in absolute and averaged values. From the data, we found that compensation in the cutting references enabled patterning the small channel within similar results as the bigger microchannel. For each particular design the error evaluation must be done considering the effect of a potential effect of a particular dimension deviation, for instance, major deviations in *r_a_*, *r_b_*, and *r_c_* could result in incompatibility for interfacing the microdevice with tubing. Among the assessed conditions, setup VI showed the overall better performance by performing single cutting over the thicker blue material using the higher available cutting load in the plotter (*f_load_* = 1 N). Hence, these settings were selected for the development of the unbalanced split micromixer of the following section.

The time required for PDMS device production is roughly 3 h (1 h for preparation and 2 h for curing) [27]. Hence, to massively develop these devices it would be necessary to have a set of master molds or to have the time required to manufacture the devices from a single mold. In contrast, a xurography-based patterning process holds the potential to sustain a manufacturing process without any molding procedure that can be developed serially within minutes for each device. Dependence on operatives for assembling the devices and bubble formation between the layers of the devices during layer stacking are some issues to be considered. Device production requires a skill set of basic non-automatic procedures. These tasks (material loading, tool handling for material formation or sealing the device) are simple and could be explained thoroughly through supporting videos and color-coded materials. For contingencies, quickness provide an opportunity for the following deployment strategies: (1) to prepare kits with pre-assembled devices and (2) to develop microfabrication stations relying on a few low-cost plotter equipment with designs from a database or digitally delivered (through cellular data signal or portable memory unit) upon requirement.

**Figure 7:**
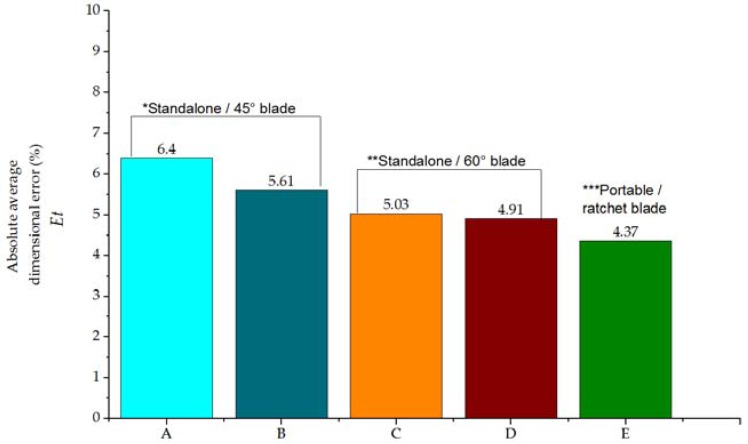
Absolute average dimensional error *E_t_* (%) for standalone and portable setups (Table 4).

Despite substitution of key components in the experimental setup (such as the blade or the plotter system) the absolute average dimensional error remains under 8% (see Figure 7). Small differences between the performance of larger standalone and smaller and portable equipment supports the feaseability of xurography for development of POC devices. Moreover, a remarkable performance (absolute average dimensional error 4.37%) suggests that the overall footprint might be more suitable for the production of medium sized designs. Process (materials, blade wear) and enviromental conditions (temperature or humidty) might impact performance, however, variations were within a manageable range.

### 4.3. Split and Recombine Passive Micromixer

A more complex passively driven micromixer was microfabricated to assess the methodology presented in this article. The design was selected pursuing to build a microfluidic device that relies their function on the geometrical features without the assistance of active pumping systems. Insights from the limits and capabilities of the quality assessment were considered to create a scaled up version of a six segment micromixer based on splitting and recombination of flow streams [34]. The design was adapted on a larger scale to be manufactured without requiring any dimensional feature compensation. The proposed design was evaluated first using CFD analysis and then evaluated qualitatively on a micromanufactured device.

Reynolds number is conventionally used to characterize the fluidic behavior in microdevices and is defined as the ratio of inertial to viscous forces. Equation (1) represents Reynolds number (Re) defined as:

(1) $$Re=\frac{inertial\;force}{viscous\;force}=\frac{\rho UD_{H}}{\mu }$$

where μ is the viscosity (Pa·s), ρ is the fluid density (kg∙m^−3^), and U is the average velocity of the flow (m·s^−1^), and D_H_ is the hydraulic diameter of channel (m) which is defined in Equation (2) as:

(2) $$D_{H}=\frac{4A}{P}=\frac{4wd_{m}}{2w+2d_{m}}$$

where *A* and *P* are the area and the wetted perimeter of the cross-section, which is given by the micromixer width (*w*) and depth (*d_m_*). The Peclet number represents the ratio between the mass transport due to convection and that of diffusion:

(3) $$Pe=\frac{advective\;transport}{diffusion\;transport}=\frac{UL}{D_{c}}$$

where *L* is the mixing path (m) and *D_c_* is the diffusion coefficient.

Fluid flows in microchannels are typically at low Reynolds number (Re < 1) due to small hydraulic diameters and relatively low volumetric flow rates, and the inertial effects (secondary flow or turbulence) are negligible [46]. A high Reynolds number above a critical value (around 2300 on microscale) indicates a turbulent flow.

To quantify the mixing behavior, the variance of the liquid species in the micromixer (σ) was calculated. The variance of the species was determined at cross-sectional area at the output of the micromixer perpendicular to the x-axis. To evaluate the degree of mixing, the variance of the mass fraction of the mixture in a cross-section (σ) that is normal to the flow direction is defined as follows:

(4) $$\sigma =\sqrt{\frac{1}{N}{\left(c_{i}-c_{m}\right)}^{2}}$$

where *N* is the number of sampling points inside the cross section, *c_i_* is the mass fraction at the sampling point I, and *c_m_* is the optimal mixing mass fraction, which is 0.5 at any cross-sectional plane (ideal mixing). To quantitatively analyze the numerical mixing performance of the micromixer, the mixing index (M_n_) at a cross-sectional plane is defined as:

(5) $$M_{n}=1-\sqrt{\frac{\sigma^{2}}{\sigma_{max}^{2}}}$$

where the mixing efficiency (ranges from 0.00 (0% mixing) to 1.00 (100% mixing). The maximum variance σ_*max*_ represent a completely unmixed condition. These values were evaluated from the planes defined at the output of each of the micromixer setup as described in the Section 3.5.2.

**Figure 8:**
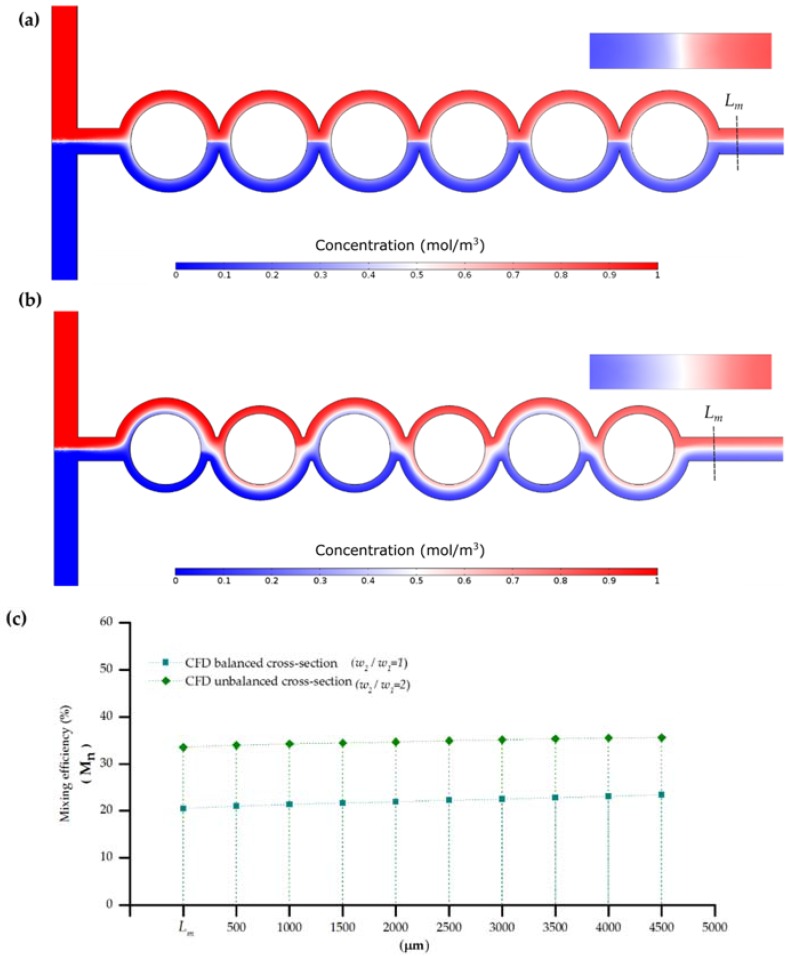
Mixing performance numerical analysis for a device with Re ≈ 0.7; (**a**) SAR micromixer (*w_2_/w_1_* = 1); (**b**) ASAR micromixer (*w_2_/w_1_* = 2); (**c**) Cross-sectional numerical mixing efficiency (*M_n_*) for (balanced SAR) and unbalanced micromixers (ASAR).

From prior experimentation, a flow rate of 0.004 m∙s^−1^ was estimated at the entrance of the micromixers. Following Equations (1) and (3) a Re of 0.7 and Pe of 130,000 can be estimated for the balanced and unbalanced configurations. The low Re and high Pe indicates that reduction in the mixing length should be carried by transversal components of flow that stretch and fold volumes of fluid over the cross section of the channel. Figure 8 shows the concentration profile at steady state for the balanced and unbalanced split and merge micromixers with a cross section formed by the microchannel width *w* = 750 μm and 100 μm of depth. Both setups were evaluated under similar boundary conditions; concentration was set to 1 (mol∙m^−3^) at the upper input and to zero at the lower input. As predicted from previous literature, the mixing efficiency for low Re is limited, as the two fluids flow adjacently tend to show no convective mixing [47]. This behavior is particularly noticeable in the case of the balanced micromixer (Figure 8a) where the concentration profile is preserved along the microchannel downstream.

In contrast, Figure 8b shows the effect of the sub-channels in the region where the interface shifts after each segment of the device. The major presence of white regions in the Figure 8b denotes completely mixed areas attributable to the effect derived from splitting and merging the interface of the stream of the main channel iteratively after each phase. Quantification of the mixing efficiency (Figure 8c) confirms the role of the design in the overall performance of the microdevice, for the current parameters the asymmetry contributes itself with around a 150% improvement comparatively with the symmetric balanced setup. Downstream the output channel it is noticeable that efficiency keeps improving by the effect of interfacial diffusion. The numerical evaluation suggests that the scaling up the dimension of the device does not hinder the ability of the device to mix the sample.

**Figure 9:**
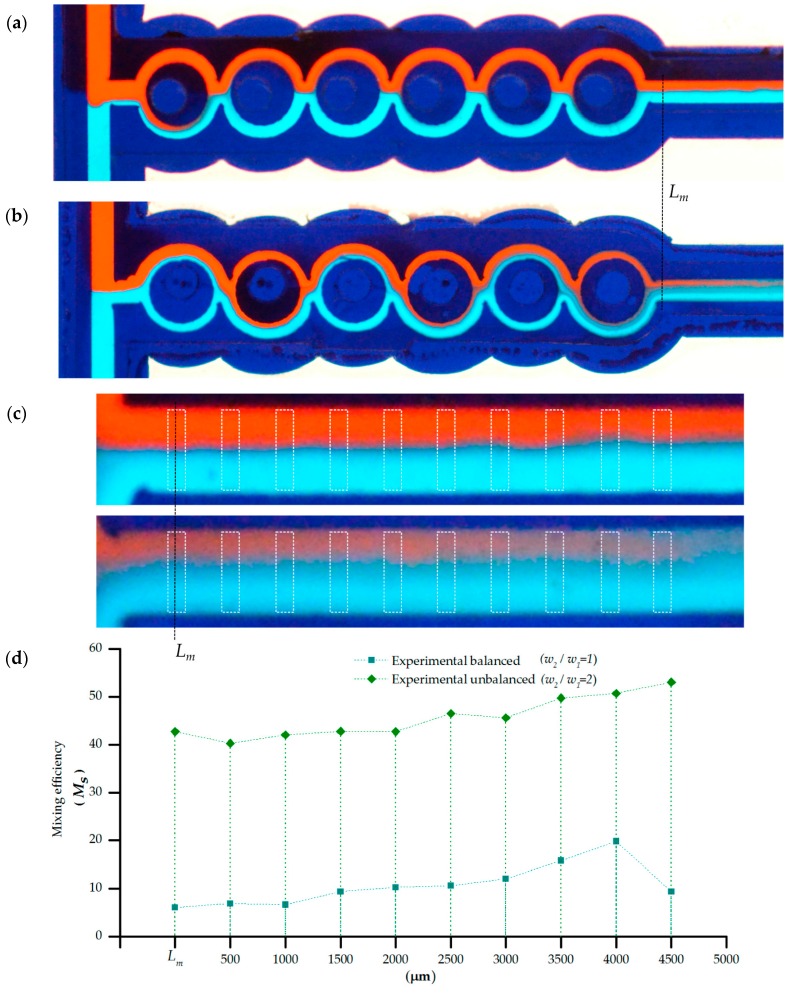
Passive micromixing in a xurography rapid fabricated microdevice for red (upper inlet) and clear blue (down inlet). The microchannels walls are conformed by the dark blue vinyl processed by xurography and lamination process; (**a**) SAR micromixer (*w_2_/w_1_* = 1); (**b**) ASAR micromixer (*w_2_/w_1_* = 2); (**c**) Windows delimited for evaluation of the experimental mixing efficiency (*M_s_*); (**d**) Experimental mixing efficiency (M_s_) at the output region of the circular based SAR and ASAR micromixers manufactured with xurography and lamination.

Figure 9a,b illustrate the mixing performance of the microfluidic devices developed using the methodology presented in this article for the balanced and unbalanced charges setups, respectively. Xurography and lamination allowed us to consistently develop devices that enclose and guide the liquid flow in a microfluidic channel formed from the integration of the different layers. After a few uses (typically 5 to 10 times), the devices became clogged in certain regions, presumably as a result of either ollapsed regions between layers along the chamber formed by the vinyl gasket of the microdevice or debris particles produced from materials from the lamination and/or assembly processes. Figure 9a shows that the interface region was kept downstream. For balanced collision of fluid streams, the interface of the fluid streams at the exit of the last mixing segment is still very clear and without any deformation. Poor mixing is shown as the streams of the fluids symmetrically split in equal volumes in the sub-channels along the mixing segments. In contrast, for the unbalanced situation Figure 9b shows a more homogenous region at the output under similar flow and sample conditions. The difference in the mixing performance is derived from the inertia of the fluid flow from the unequal widths in the split channels. The geometrical features provoke collision between the fluid stream which perturbs the flow and shifts the interface of the fluid streams into the sub-channel where Dean vortices become effective in enhancing mixing [34,35]. Experimental observation on the mixing capability of the device was consistent with the CFD simulation showing a better performance for the unbalanced case. To estimate surface mixing performance, the mixing index (*M_n_*) at the output of the micromixers was evaluated using ten evaluation windows (Figure 9c).

Mixing was characterized by the variance in the intensity of color component as described in Section 3.5.3 using the following equation:

(6) $$M_{s}=1-\sqrt{\frac{{\sigma_{w}}^{2}}{\sigma_{max}^{2}}}$$

Figure 9d shows, in agreement with the numerical study, better performance for the ASAR setup. From the examination of the detail of the image of Figure 8c it is noticeable that downstream there is an incremental performance of the mixing. It is possible that this behavior is biased by our quantification window (susceptible to disturbances caused by imperfections on the walls) or noise from the detection system. A linear fitting for the experimental data showed that mixing performance for the symmetric balanced configuration could correspondingly be approximated to 11% and 46%.

It is noticeable that the mixing efficiency for the featured microdevices is limited by the small impact of inertial forces at regimes with low Reynolds numbers. Unbalanced collision of fluid streams improved the mixing performance as compared to balanced collision accordingly with the simulation presented in this article and numerical analysis from the literature review. The performance can be explained considering that while Dean vortices are present in the sub-channels of both setups, for the unbalanced micromixer collisions between the fluid streams shift the interface between them into the sub-channels where Dean vortices become effective in mixing [34]. Hence, the difference in performance between balanced and unbalanced micromixers is due to the geometrical variations and therefore derived directly from the manufacturing process.

Moderating the flow rate through the addition of some active pumping system could provide better mixing performance due to the possibility of obtaining better regime conditions for micromixing. Moreover, characterizing the flow from passive pumping (for example by controlling the flow through surface tension) could be adopted more easily in developing countries for disposable devices. Increasing the flow rate passively and the number of mixing segments could provide a feasible and economic mixing solution for some sensing applications.

Differences between experimental and computational results are likely arisen due differences in the properties of the materials used in the model and the experimental values from the samples utilized in the device during experimentation.

## 5. Conclusions and Future Work

The study presented here shows the viability of the xurography process for rapid fabrication of microfluidic Point-of-Care (POC) devices. The main benefits found can be summarized as follows:

- Xurography provides a wide range of manufacturing flexibility, without the assistance of specialized equipment or facilities.
- In terms of product quality, absolute average dimensional errors below 8% can be achieved.
- The cycle time for design and manufacture of POC devices is on the order of days. For example, the study shows the manufacture of geometric feature-dependent devices (splitting and merging micromixer) in a relatively short period.
- A short cycle time, and associated cost, makes xurography suitable for disposable POC devices.

Further improvements in xurography-based micromixing technology could be achieved by additional research in terms of: (a) tunable and controlled pumping systems; (b) performance monitoring with sensors; and (c) modeling, calibration and compensation for systematic manufacturing errors.

## Figures and Tables

**Table 1:** Applications employing xurography and lamination as a manufacturing technology.

Work (year)	* Ref	Function	Manufacture	** Materials	Assembly
Weigl *et al.* (2001)	[9]	Cytometry and H-filter chambers	CO_2_ laser cutting, oxygen plasma treatment, lamination	PET sheets	Metal frame housing/double sided adhesive
Do Lago *et al.* (2003)	[10]	EP flow chamber/Electrospray tip	Laser printing, drilling, Gluing, Scissor cutting, lamination	Toner PET (acetate sheet)	Thermal lamination
Bartholomeusz (2006)	[7]	Shadow mask, PDMS micromolding, coiled channel	Xurography, lamination, Sputtering	Rubylith, Vinyl, Polyester, Aluminium, Sandblast, glass	Thermal lamination
Greer *et al.* (2007)	[11]	DNA analysis well	Xurography, drilling and heat treatment	Double sided tape, glass slides, PEEK (Nanoport)	Adhesive, thermal bonding
Sundberg *et al.* (2010)	[14]	PCR disk platform	Xurography, Thermal lamination	PETG sheets, PTFE strips	Thermal lamination
Santana *et al.* (2013)	[13]	Mask for glass etching process (EP chamber)	Xurography	Vinyl	-
Kim *et al.* (2014)	[15]	Electrochemical biosensing	Xurography, Au sputtering, CO_2_ laser cutting	PET, double-sided tape, PMMA, Au	Cold lamination (machine)

* Reference; ** Device conforming materials.

**Table 2:** Cutting parameters assessment conditions for quality optimization (Layer 1).

Setup	Nominal Microchannel Width *w_nom_* (μm)	Nominal Microchannel Wall Width *w_wall_* (μm)	Cutting Load *f_load_* (N)	Cutting Passes *N*	Material Nominal Depth *d_nom_* (μm)
I	750	500	0.8	2	75
II	750	500	1.0	1	75
III	750	500	1.0	1	50
IV	750	500	0.8	2	50
V	200	500	0.8	2	75
VI	200	500	1.0	1	75
VII	200	500	0.8	2	50
VIII	200	500	1.0	2	50

**Table 3:** Nominal geometric features dimensions of T-micromixer.

Geometric Feature	Setups I to IV, Nominal Dimension (μm)	Setup V to VIII, Nominal Dimension (μm)
*w_a_*	750	200
*r_a_*	1000	1000
*w_b_*	750	200
*r_b_*	1000	1000
*w_c_*	750	200
*r_c_*	1000	1000
*w_x_*	750	200
*w_y_*	750	200
*w_z_*	750	200

**Table 4:** Cutting performance comparison with other setups.

Setup	Plotter	Blade	Patterning Parameters
*A*	Graphtec CE5000-60	CB09U (45°)	f_load_ ≈ 0.8 N, *N* = 1
*B*	Graphtec CE5000-60	CB09U (45°)	f_load_ ≈ 0.53 N, *N* = 2
*C*	Graphtec CE5000-60	CB09UA-1 (60°)	f_load_ ≈ 0.8 N, *N* = 1
*D*	Graphtec CE5000-60	CB09UA-1 (60°)	f_load_ ≈ 0.6 N, *N* = 2
*E*	Silhouette Portrait	Ratchet 3-3T	Blade depth = 3, material depth = 5

**Table 5:** Layer by layer composition and function.

Layer	Material	Function
*0*	PMMA	Substrate
*1*	Vinyl	Formation of flow cell walls
*2*	Acetate sheet	Formation of flow cell ceiling and delimitation of inlets and outlets
*3*	Translucent vinyl	Sealing and delimitation of inlets and outlets

**Table 6:** Cutting parameter compensation for setups V to VIII.

Reference	Nominal Dimension (μm)	Compensation (μm)
*w_a_*	200	+55
*w_c_*	200	+60
*w_x_*	200	+55
*w_z_*	200	+60

## Supplementary Materials

The following are available online at www.mdpi.com/1424-8220/16/5/705/s1, Video S1: Sealing test of a T-micromixer.

## Abbreviations

The following abbreviations are used in this manuscript:

ASAR: Asymmetric split and recombination
CAD: Computer aided design
CFD: computational fluid dynamics
COC: Cyclic olefin copolymer
EP: electrophoresis
SAR: split and recombination
SGM: slanted grooved mixer
SHM: staggered herringbone mixer
LOC: Lab-On-a-Chip
PC: polycarbonate
PDMS: polydimethylsiloxane
PEEK: polyether ether ketone
PET: polyethylene terephthalate
PETG: glycol-modified polyethylene terephthalate
PMMA: polymethyl methacrylate
PETG: polyethylene terephthalate glycol
RGB: red-green-blue
PCR: polymerase chain reaction
POC: Point-of-Care
WHO: World Health Organization
